# SpotLight Proteomics: uncovering the hidden blood proteome improves diagnostic power of proteomics

**DOI:** 10.1038/srep41929

**Published:** 2017-02-07

**Authors:** Susanna L. Lundström, Bo Zhang, Dorothea Rutishauser, Dag Aarsland, Roman A. Zubarev

**Affiliations:** 1 Division of Physiological Chemistry I, Department of Medical Biochemistry and Biophysics, Karolinska Institutet, Stockholm, Sweden; 2 Alzheimer’s Disease Research Centre, Department of Neurobiology, Care Sciences and, Society, Karolinska Institutet, Stockholm, Sweden; 3 Centre for Age-related diseases, Stavanger University Hospital, Stavanger, Norway; 4 Department of Old Age Psychiatry, Institute of Psychiatry, Psychology & Neuroscience, King’s College London, London, United Kingdom

## Abstract

The human blood proteome is frequently assessed by protein abundance profiling using a combination of liquid chromatography and tandem mass spectrometry (LC-MS/MS). In traditional sequence database search, many good-quality MS/MS data remain unassigned. Here we uncover the hidden part of the blood proteome via novel SpotLight approach. This method combines *de novo* MS/MS sequencing of enriched antibodies and co-extracted proteins with subsequent label-free quantification of new and known peptides in both enriched and unfractionated samples. In a pilot study on differentiating early stages of Alzheimer’s disease (AD) from Dementia with Lewy Bodies (DLB), on peptide level the hidden proteome contributed almost as much information to patient stratification as the apparent proteome. Intriguingly, many of the new peptide sequences are attributable to antibody variable regions, and are potentially indicative of disease etiology. When the hidden and apparent proteomes are combined, the accuracy of differentiating AD (n = 97) and DLB (n = 47) increased from ≈85% to ≈95%. The low added burden of SpotLight proteome analysis makes it attractive for use in clinical settings.

In recent years, quantitative proteomics has developed rapidly, offering clinical analyses of blood serum and plasma at relatively low cost and high throughput. Two approaches are generally used: one utilizes antibodies^1^,^2^, and the other method uses a combination of nano-flow liquid chromatography and tandem mass spectrometry (nLC-MS/MS)^3^,^4^. Both approaches make use of *a priori* known information: antibodies are developed against common proteins and/or their known posttranslational modifications (PTMs), while the LC-MS/MS approach for protein identification matches MS/MS spectra against a database of known sequences, taking only a few common PTMs into consideration. Even though these approaches have proved their utility in a large number of studies, they both miss unknown or unexpected sequences and PTMs. This missing information may be important, or even crucial, for building proteome-based diagnostic and prognostic models and for understanding the disease origin and progression.

A decade ago, we have analyzed proteomics data obtained with at that time most advanced instrumentation available, featuring high-resolution MS combined with high-resolution MS/MS employing two complementary fragmentation techniques^5^. Despite the excellent data quality, it was found that 25–30% of the good quality MS/MS-data still don’t match the database sequences^6^. The root of the problem was hypothesized to be the presence of unexpected PTMs, mutations and altogether new sequences. In order to address the issue of the wide and *a priori* unknown repertoire of PTMs present, the untargeted ModifiComb approach to PTM analysis was introduced^7^. Other groups have pursued similar approaches^8^. Note that, from the standpoint of an unbiased PTM analysis that deals with PTMs of both positive and negative mass shifts, there is no difference between a PTM and a mutation. Usually, approaches such as ModifiComb detect PTMs and mutations that do not alter the sequence too much. However, new sequences may also be present in the proteome due to carry-over between heterogeneous samples or potentially from contaminations by virus, bacteria or mycoplasma^9^. In analysis of clinical samples, the *de novo* sequencing proteomics approach can provide information on disease-specific polymorphism in proteins. In particular, the *de novo* sequencing approach may identify disease-related differences due to the intrinsic sequence heterogeneity of native antibodies (Immunoglobulins, Igs) in patient blood. Theoretically, antibody recombination and point mutations can result in over 10^15^ different antigen-binding sites in humans^10^. However, human antigen response only exploits ∼1% (10^13^) of this sequence diversity^11^,^12^,^13^. Yet, this number is still 10^10^ times bigger than the number of proteins in the remaining blood proteome, and the *a priori* probability to detect by MS an antibody molecule with a given sequence is vanishingly small. However, recent studies have revealed that antigen specific antibody homology is more frequent than would be expected by pure chance^14^,^15^,^16^,^17^,^18^,^19^,^20^,^21^. Indeed, when the immune system in different individuals is challenged by the same antigen, the antibodies raised against this challenge should bind to it efficiently, which puts restraint on sequence variability of these antigen-specific Igs. In a homogeneous group of patients, the abundance(s) of peptides from the homologous Ig variable region with binding affinities to disease-specific antigen(s) may even be high enough to be detected by MS and may correlate with the disease strongly enough to be useful as biomarker(s). Since the Ig sequences of interest are unlikely to be found in standard sequence databases, analysis of the hidden blood proteome requires *de novo* polypeptide sequencing.

Here, we introduce the SpotLight approach to the analysis of the hidden blood proteome. Given that a majority of polymorphism within the blood proteome is derived from antibodies, the SpotLight approach includes a simple enrichment step for polyclonal Immunoglobulin G (IgGs) using Melon Gel (MG). MG enrichment is not based on Fc-region specificity and certain blood proteins (herein referred to as MG proteins) are also co-enriched.

To produce and annotate a database of IgG and other *de novo* sequences, SpotLight employs several important steps prior to regular standardized label-free proteomics database search and quantitation (Fig. 1). The MG-enriched fraction is digested and analyzed by LC-MS/MS using two complementary fragmentation techniques. Both MS and MS/MS data are acquired with high resolution, which is a pre-requisite for reliable *de novo* sequencing^22^. Newly derived sequences are analyzed by BLAST in terms of homology to either IgG or other proteins. In any case, their sequences are inserted in the sequence database, together with the tentative assignment. Next, the LC-MS/MS datasets of both intact and MG-enriched proteomes are processed using our novel DeMix-Q label-free workflow for peptide identification and quantification as well as for protein inference^23^,^24^.

To test the SpotLight approach, we selected a cohort of early stage patients diagnosed with similar neurodegeneration disorders: Alzheimer’s disease (AD) and Dementia with Lewy Bodies (DLB). These disorders have similarities in pathology, and their differentiation is nontrivial^25^ but important due to the differences in prognosis and treatment response. Clinical criteria have good specificity but relatively low sensitivity, and there is a need for accurate, cheap, and easily available biomarkers. Due to overlap in pathology, CSF and MRI-based biomarkers are not sufficiently accurate^25^. The 144 patients (97 AD and 47 DLB; see Table 1) were separated into a homogeneous Group A (24 AD and 24 DLB) that was used for multivariate (MV) statistical analysis and model building, and a heterogeneous Group B (remaining patients) that was employed for model verification. Subsets of the generated data, (i.e. intact and MG-enriched proteomes; proteins and peptides, IgG and non-IgG peptides; known and new peptides) were tested according to the quality factor Q^2^ of the model, the p-value of AD/DLB separation and the area under the Receiver Operating Characteristic (ROC) curve, AUC. The results revealed the potential for disease diagnostics of the hidden proteome, manifested in its high predictive power.

## Results

### IgG-isolation

We searched for a simple and robust way to enrich human serum IgGs, eventually selecting Melon™ Gel IgG Spin Purification Kit (Thermo Fisher Scientific). Contrary to Protein G and Protein A IgG-purification, MG enrichment is not based on Fc-region specificity, but instead on gel binding of non-IgG proteins, thereby allowing IgG recovery in the flow-through (non-bound) fraction. Certain blood proteins (i.e. MG proteins) were found co-enriched with the IgGs. Their abundances did not correlate with those in intact serum (see below). Some MG proteins might be enriched because of the immune complex formation with IgG, or because of the similarities in their physico-chemical properties to IgG (pKα values, etc.).

### *De novo* sequencing and database expansion

The MG-enriched and intact serum samples were digested by trypsin and analyzed by LC-MS/MS. To obtain reliable *de novo* sequencing of tryptic peptides, both higher-energy collisional dissociation (HCD) and electron transfer dissociation (ETD) MS/MS were used, and the fragments were detected with high resolution. In parallel, intact blood serum digests were analyzed using high resolution HCD MS/MS only. Each HCD-ETD MS/MS spectral pair from the MG-enriched samples was submitted to PepNovo+ for *de novo* sequencing^26^. Up to nine best candidates representing full backbone coverage were accepted for every HCD-ETD MS/MS dataset. The candidate sequences were searched against the UniProt protein database using BLASTp (Protein BLAST). The sequence candidate with the highest BLAST score was reported as the final sequence for a given HCD-ETD MS/MS spectral pair. The identity was reported as “protein” or “IgG”. The UNIPROT protein sequence database was then expanded by adding the new sequences. The novel DeMix-Q workflow^23^,^24^ was used to perform peptide identification in the expanded database and quantification of the intact and MG-enriched proteomes. As protein abundance, the sum of the abundances of three most abundant known peptides was taken.

### Uncovering the hidden proteome

In the intact (unfractionated) serum proteome, 156 proteins were quantified, while 81 proteins were quantified via MG-enrichment. Of these, 23 proteins were new (not found in the intact proteome). On the peptide level, the intact proteome contained 2112 unique sequences (of which 467, or 18%, were related to IgG), while the MG-enrichment proteome contained 2077 peptides, of which 646 (31%) were homologous to IgG peptides. Note that only data from peptides (and proteins) that were robustly quantified in at least 50% of all AD or 50% of all DLB patients were considered. Overall, for both the total number of quantified peptides (n = 3019) and the filtered peptides (n = 2077) the sensitivity to *de novo* sequenced peptides was as good as for those obtained via conventional database search (Supplementary Fig. 1).

The contribution of *de novo* sequencing to the analysis was significant (Supplementary Fig. 2). Of the 2112 non-IgG peptides quantified in the intact proteome, 435 (21%) were from novel sequences. Even bigger was the impact on the MG-proteins: 706 non-IgG peptides out of 1431 sequences (49%) were derived by *de novo* sequencing.

The abundances of the ∼40% (n = 610) of the non-IgG peptides that were quantified in both proteomes correlated only weakly between the proteomes (R^2^ = 0.2, Supplementary Fig. 2C), thus confirming that the two proteomes provide largely complementary information. On the other hand, the peptides homologous to the heavy variable (HV), kappa variable (KV) and lambda variable (LV) chain regions of IgG showed as expected an improved significant (p < 0.0001) correlation between the two proteomes (R^2^ = 0.6, Supplementary Fig. 2B). The majority of these peptides (280) were identified by *de novo* sequencing compared to 72 sequences obtained by database matching. This four-fold increase in characterized IgG sequences represents a dramatic expansion in information content, rendering the “IgGome” to be a separate subdomain of the total proteome. Altogether, almost 2000 IgG-related peptide sequences were identified, but most of them were not quantified in a majority of patients composing one group, and thus discarded from the analysis. It is likely that in some samples the peptides in question were actually present, and that the main reason for their non-detection was the limited dynamic range of MS. As the sensitivity of the MS instrumentation as well as the MS/MS efficiency improve with time, the relative importance of the IgGome in respect to the intact proteome is very likely to increase.

Of the quantified new sequences, 120 peptides originated from the complementary determining (CDR) regions (Supplementary Table 1). For annotation of the peptides to proteins, we used BLAST search, which incurred possible limitations. Thus, only four longer (>5 AA residues) novel sequences were identified as homologous to the CDR3 region, which is the most diverse part of the IgG molecule. The CDR3 peptides sequenced *de novo* and showing little or no homology to known sequences were likely to be classified in our approach as non-IgG peptides.

Yet, the fact that ∼100 *de novo* sequenced CDR peptides were quantified in a majority of patients testifies to the validity of the hypothesis postulating high degree of homology in variable regions of abundant antibodies in homologous patient groups. The abundances of these peptides can be used for differentiating between the two diseases (see below).

### Differentiating between AD and DLB

To evaluate the impact of the hidden proteome on the ability of proteomics data to differentiate between AD and DLB at the moment of diagnosis, MV-analysis of peptide and protein abundances was performed. In a conservative approach, patient samples were split into two groups. The homogeneous Group A contained 24 AD- and 24 DLB-patients, balanced in terms of gender and age (Table 1). The data from Group A were used in the Orthogonal Projections to Latent Structures-Discriminant Analysis (OPLS-DA) approach^27^,^28^ to build a model for AD/DLB differentiation. This model was then applied to the heterogeneous Group B encompassing the remaining patient samples (AD n = 73, DLB n = 23), and for additional testing, nine patients with Parkinson Disease (PD) (Table 1). Cross-validation was used to evaluate the model, and the area under the ROC-curve (AUC) assessed the predictive power of the underlying data.

**Figure 2:**
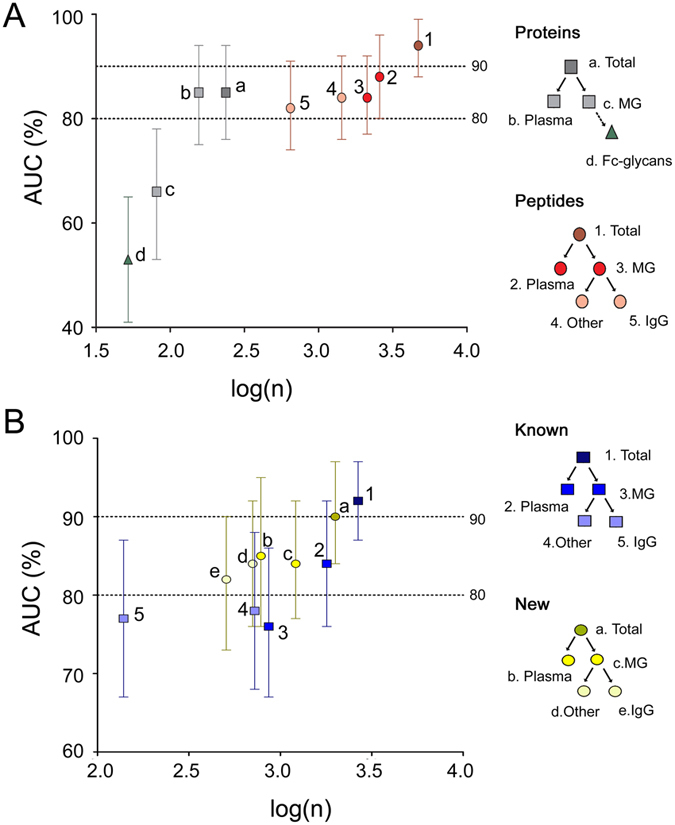
AUC of AD/DLB disease discrimination of Group B patients (treated as unknown) against the logarithmic number of proteins/peptides included in respective MVA model based on Group A patients. (**A**) Based on proteins, peptides and Fc-glycans. (**B**) Based on known and new (*de novo* sequenced) peptides. Error bars represent 95% confidence intervals. Total: all variables, Plasma: Plasma extracted molecules, MG: Melon Gel extracted molecules.

Similar to our previous experience^29^, the model based on 156 proteins from the intact proteome gave an AUC of 85%, while the model based on the 81 MG-enriched proteins generated a low AUC of 66% (Fig. 2A). The combined model of both the intact and MG proteins also gave an AUC of 85%. Therefore, at the protein level the hidden proteome did not yield any noticeable improvement.

**Figure 3:**
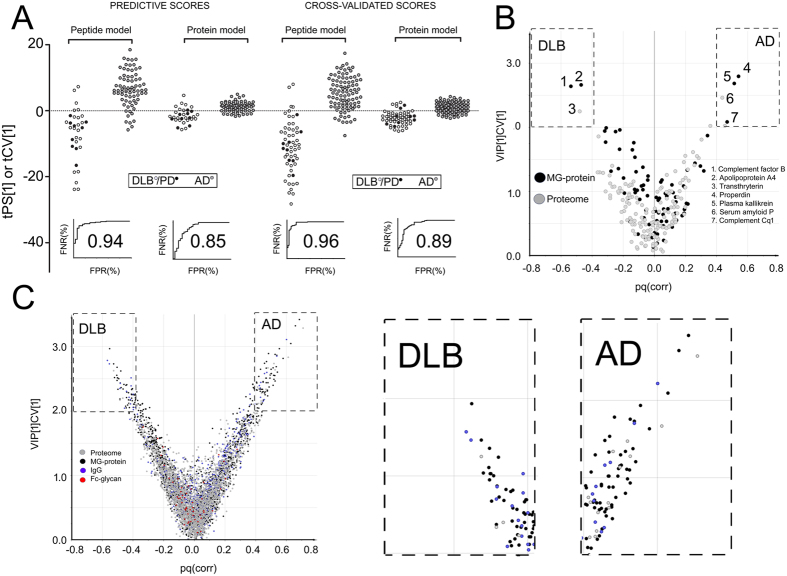
Multivariate combined models of the predictive scores of Group B and cross validated scores of Group A and Group B combined. (**A**) Left, Predicted scores (tPS) of the Group B- and PD-patients (all treated as unknowns) in the combined OPLS-DA models (peptides and proteins) based on the differences in the AD- and DLB Group A patients. Right, the cross validated scores (tCV) of the combined OPLS-DA models (peptides and proteins) based on all patients. The predictive scores of the PD patients (still treated as unknowns) are also included in the plot. The corresponding ROC-curves of each model are shown below the compared patients. (**B**) Biomarker selection from the combined patient protein model using Variable Influence in Projection (VIP) values plotted against the p(corr) values. (**C**) Biomarker selection from the combined patient peptide model using Variable Influence in Projection (VIP) values plotted against the p(corr) values. Loading plots for proteins and peptides that correlated with >99% confidence with each sub-domain are given in Supplementary Table 3.

However, at the peptide level, the AUCs improved for all models (Figs 2A and 3A and Supplementary Table 2). The AUC for the 2579 intact proteome peptides was 88%, for the MG peptides (n = 2129) it was similar (84%), and for the combined model the AUC went up to 94%. Separately, 1431 MG-enriched non-IgG peptides gave an AUC of 84%, while the 646 IgGome peptides alone provided 82%.

Despite their lower number compared to the 2659 database-matched peptides, the 1997 *de novo* sequenced peptides demonstrated high predictive power, with an AUC of 90% (Fig. 2B and Supplementary Table 2). In general (and as expected) the prime factor determining the model accuracy is the number of molecules included in the model (Fig. 2). Hence, the combined peptide model was the most accurate, while the smallest model (52 Fc-glycans) was the least accurate. The largest deviations from this trend are the models for proteome proteins (n = 156, AUC 85%) and the new *de novo* sequenced peptides of the intact proteome (n = 784, AUC 85%), MG-proteins (n = 706, AUC 84%) and the IgGome (n = 507, AUC 82%) (Fig. 2 and Supplementary Table 2). However, doubling the number of sequenced peptides via *de novo* sequencing would not be useful unless many of these molecules possess as much (or more) predictive power as traditionally sequenced peptides.

In a less conservative approach, Groups A and B data were merged, and unified OPLS-DA models of AD/DLB separation were built on the peptide and protein levels with subsequent cross-validation (Fig. 3). Generally, this approach yields a slight overestimation of the predictive power of the model and the underlying data and thus a higher AUC value. Hence, for extra validation the PD patients were tested on the models as predictors.

For the all-patient model that included the data on both the intact and MG-enriched proteomes, an AUC of 89% was obtained for proteins and of 96% for peptides (Fig. 3A). Taking the average between the conservative (Group A) and “all-patient” models, generates an estimated predictive power for peptides of 95%.

Figure 3B and C show which molecular entities contributed most to the predictive power of the models. For each entity, its relative importance for the model (y-axis, Variable Influence in Projection (VIP-CV)) is plotted against its correlation with the disease type (x-axis, p(corr))^28^. Both the proteome and MG entities are contributing to the DLB/AD-differentiation (dashed boxes in the plots). Among the 514 peptides that correlated with either DLB or AD with ≥99% confidence, there are three Fc-glycopeptides, 63 peptides from the IgGome (75% of them - *de novo* sequenced), 250 from MG (43% *de novo* sequenced) and 198 peptides from the intact proteome (33% *de novo* sequenced) (Supplementary Table 3). In the model based on proteins, eight MG proteins and three intact proteome proteins correlated with either patient group with ≥99% confidence (Supplementary Table 3).

Of notice, non-supervised Principle Component Analysis (PCA) also indicated separation between the two patient groups in particular components. In Supplementary Fig. 3 we show the PCA model scores of the model that was based on the complete data set and which included all patients (R^2^ = 0.51, Q^2^ = 0.21, 23 components). The figure shows component 3 and component 5 for which the best separation between the groups was observed.

### Potential biomarkers of AD and DLB

Using the most conservative approach to statistical significance, we applied Bonferroni correction (BF, n = 4945) to the p-values. In the intact proteome, four proteins (transthyretin, serum amyloid P component, apolipoprotein D and multiple PDZ domain protein) and 47 peptides were found at significantly different levels in the two diseases, of which 13 molecules were identified via *de novo* sequencing (28%). Of the significant peptides, 18 (∼40%) originated from the proteins that also had significantly different abundances (with or without BF-correction) (Supplementary Table 4). Consistent with previous AD-biomarker studies^30^,^31^,^32^, transytherin had a lower abundance in AD-patients, while serum amyloid P component had a higher abundance (Table 2).

Plasma kallikrein, properdin and complement C1q subcomponent subunit B (C1qb) were significantly elevated in the AD MG-enriched proteome (Table 2). The same phenomenon has previously been observed in AD-patient plasma and in the contact system in AD-mouse model/wild-type mice injected with Aβ42^33^. Furthermore, an increased plasma kallikrein activity has been found in AD-brain parenchyma^34^. Noteworthy is the fact that properdin has been linked to brain disorders, via polymorphism^35^,^36^,^37^. The high abundance of both C1qb and properdin may also be linked to complement pathways upregulation.

Forty-nine MG peptides had significantly different abundances in AD vs DLB, with 23 peptides (47%) originating from proteins with significantly (with or without BF-correction) different abundances (Supplementary Table 4). Of the remaining significant peptides, five *de novo* sequenced molecules were of particular interest (Table 2). PGSVFPLADVGGK, which is overrepresented in the AD samples (p = 3.8E-6, BF-corrected p = 1.9E-2), is homologous to the peptide PDSVFPLEGASDADVG from the Protocadherin-α family which is involved in brain structure and function^38^,^39^. This novel peptide is also found to be significantly elevated in the AD cohort in the intact proteome (Table 2). Additionally, two pairs of AD-elevated peptides identified by *de novo* sequencing in the MG-enriched proteome showed close sequence homology in-between the pairs, but had low sequence homology to anything else, thus indicating that they may originate from the IgG CDR3-regions (Table 2).

In the intact proteome, no peptides from the variable IgG regions were significanty different in AD vs DLB, while MG-enrichment provided eight such peptides. Of the total 13 significant IgGome peptides (one HV-, six KV-, one LV- and five conserved-chain peptides, Supplementary Table 4), 10 (77%) were identified via *de novo* sequencing and one (SSQSVLYSSNNK) matched the database sequence of the CDR1 KV-region. Interestingly, the significant HV peptide (NTLYLQMGNSLR, Table 2), was significantly elevated in AD, while its nine homologous peptides were all elevated (significantly without BF-correction) in the DLB cohort (Supplementary Fig. 4).

Examples of AUC scores of potential biomarker candidates of AD/DLB differentiation are shown in Supplementary Fig. 5.

### Impact of the ApoE genotype

**Figure 4:**
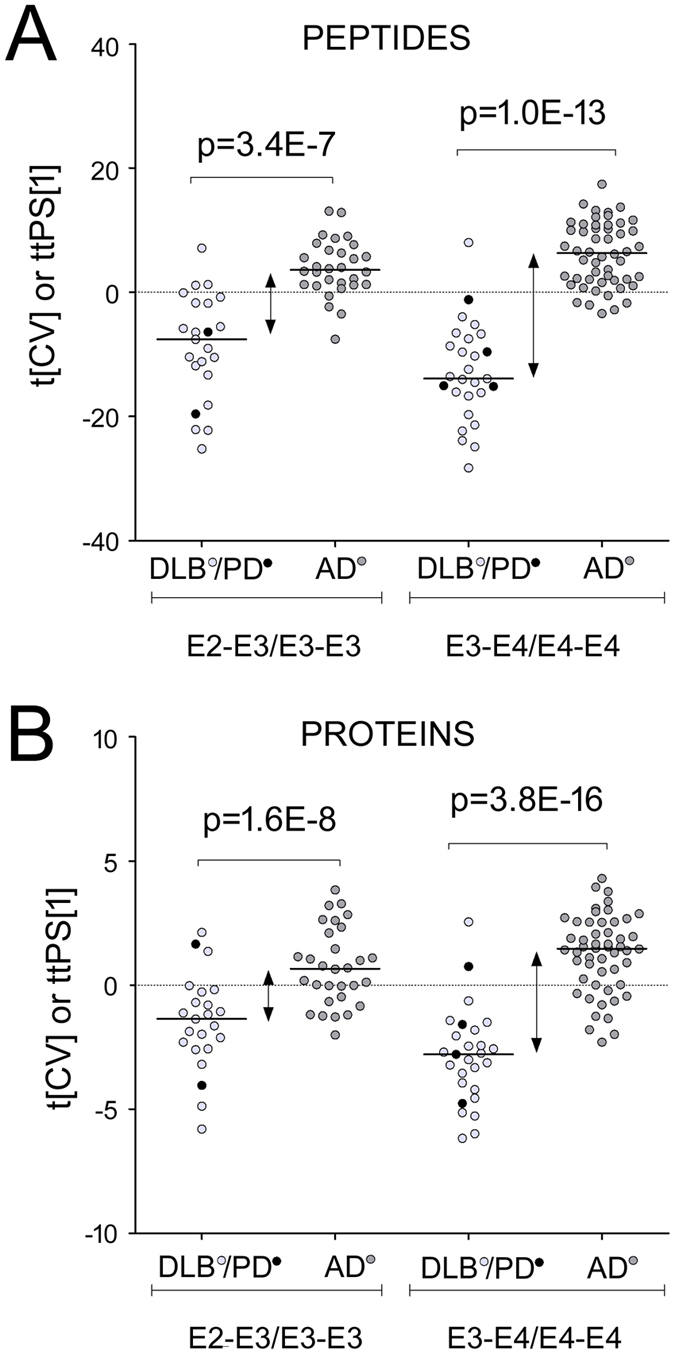
(**A**) The Cross-Validated scores from the multivariate combined peptide-based model of the complete dataset (Fig. 3A). Patients are divided according to known Apolipoprotein E genotypes (E2-E3 and E3-E3, n = 51 versus E3-E4 and E4-E4, n = 75) and disease type. (**B**) The Cross-Validated scores from the multivariate combined protein model of the complete dataset (Fig. 3A), divided according to Apolipoprotein E genotypes and disease type. Marked out lines in the plots represent the medians.

The ApoE4 isoform has an arginine residue in the position 112 instead of the cysteine residue in that position in ApoE2 and ApoE3. The tryptic peptides LGADMEDVR (ApoE4) and LGADMEDVCGR (ApoE2, ApoE3) differentiating these two alleles were detected in our datasets but not reliably quantified. However, the p-values of the MV models built using only E4-gene carriers’ data are several orders of magnitude lower than those of the models based on other patients (Fig. 4). This observation was consistent for all data domains. Thus, the confidence in distinguishing AD and DLB E4-genotype carriers were greater (p = 10^−3^–10^−20^) compared to distinguishing the AD and DLB non-E4 genotype carriers (p = 10^−2^–10^−7^) (Supplementary Table 5). Furthermore, for all new peptide sequence models of AD, the E4-gene carriers were significantly different in tCV scores (p < 2.5E-02) compared to non-carriers (Supplementary Table 5). Particularly different were the abundances of IgG peptides (both new and known sequences, as well as the Fc-glycans). Two IgG peptides in Table 2, NTLYLQMGNSLR and SSQSVLYSSNNK, were significantly elevated for the E4-genotype compared to non-carriers both in AD patients as well as in all patients combined (both - p = 0.003).

On the protein level, no molecule in either proteome was significant following the BF-correction. This does not necessarily mean that there are no differences between the patient groups on the proteome level. Using BF-correction improves the confidence in eliminating false positives, but it is likely that this will also remove in the process a number of true positives). For example, Apolipoprotein E had a significantly lower abundance for the E4-genotype not only in AD patients (p = 0.001), but also in all patients (p = 0.0003). In E4-carrying DLB patients, Apolipoprotein E levels were also lower than in non-carriers, but with a lower significance (p = 0.07). These results are in line with the previous studies that have shown that APOE-E4 carriers have lower levels of total Apolipoprotein E^40^,^41^.

## Discussion

The SpotLight approach combines two novel features, MG-enrichment and BLAST-filtered *de novo* sequencing, and greatly enhances the diagnostic power of the classical blood proteomics analysis^3^,^4^. At the modest expense of an additional (fast and inexpensive) sample preparation step and doubling the instrumental analysis time, two new domains of analysis are added – MG-enriched proteins and IgG peptides. As a result, the number of quantified peptides is almost doubled, and the predictive power of separating two common types of dementia, AD and DLB, increased from ∼85% to ∼95%. Note that this result was achieved even in a conservative approach on an altogether different subset (n = 96) of patients than the smaller subset (n = 48) used for model training. Furthermore, we also had access to nine additional samples from PD patients which had similar pathophysiology to DLB patients^25^. We used these patients as an extra control and could confirm that also on the proteome/peptidome level the PD profiles resembled those of the DLB patients. However, due to the low number of PD patient samples, we could not verify potential features that may signify a specific “PD” profile (different from both AD and DLB). As an additional validation of our method, a strong influence of the ApoE genotype in the patient’s blood proteome was found, potentially indicating four different disease related phenotypes: DLB-E4+, DLB-E4−, AD-E4+ and AD-E4−.

The most intriguing group of *de novo* sequenced peptides was from the variable CDR regions of IgGs. Of these peptides, the majority was from the CDR1 and CDR2 areas of the LV and KV chains and had a close homology to the germline sequences. Despite this homology, the abundances of one CDR1 KV peptide and several *de novo* sequenced peptides from the conserved framework regions were distinctly different between the patient groups (p-values: 10^−11^–10^−6^, Supplementary Table 4), thus indicating that the changes in the polyclonal IgG-repertoire are to a certain degree orchestrated in individuals whose immune systems are challenged in a similar way. This observation may have implications for future biomarker discovery studies. Conventional antigen-based biomarker discovery often has limited success in bodily fluids due to the issues related to large dynamic range, varying degree of excretion into the circulation, rapid turnover and overall inter-individual variability^42^,^43^,^44^,^45^. In search for an antigen (which might be elusive), discovery of the marker antibody peptides may be the first step of pragmatic importance. While the presence of an antigen in the body can be organ-specific, uneven in time and short-lived, antibody levels in blood are likely to be more persistent. Furthermore, disease-specific antibodies are usually present at higher concentrations, and can be enriched further by immunoprecipitation.

In addition to peptides with sequence homology to IgG, the MG-extracts contained hundreds of peptides with little sequence homology to known human proteins. While some of these sequences might come from sample preparation (via impure trypsin or Melon Gel) and microorganism contamination, it is among this peptide pool that CDR3 sequenced peptides are likely to be hiding. Two pairs of such peptides with close sequence homology between themselves were significantly elevated in the AD vs the DLB patients (Table 2). This part of the hidden proteome is the most intriguing, and requires special attention.

In conclusion, since blood proteomics is rapidly gaining ground in clinical setting, its enhanced version presented herein is likely to find clinical applications, e.g., in diagnosis, prognosis, drug action monitoring, and in mechanistic disease studies. Being implemented in several diseases, the SpotLight approach to proteomics can significantly expand our knowledge on selection of polyclonal IgG-repertoire in disease development and progress. This approach may further improve the specificity and accuracy of predicting the disease status in individual patients.

## Methods

### Patients

This study was conducted according to the guidelines laid down in the Declaration of Helsinki. All procedures involving human subjects were approved by the Norwegian regional Ethics Review Board (approval number 2010/633) and the Norwegian authorities for collection of medical data. Written informed consent was obtained from all subjects. Subjects were recruited into the DemWest cohort as previously described^46^. Briefly, 47 patients diagnosed with mild Dementia with Levy Bodies (DLB, age: 76 ± 7 years, 20 females) and 97 patients diagnosed with mild Alzheimer’s disease (AD, age: 75 ± 8 years, 70 females), were included in the study (Table 1, Supplementary Table 6). In addition to clinical and biomarker diagnostic procedures, patients were recruited for brain donation. For the first 46 cases analyzed post-mortem, the accuracy of the AD/DLB diagnostics was >85%. The patients were divided into two groups (Group A and Group B, Table 1, Supplementary Table 6). The Group A samples were used to generate a disease-differentiating model, which was then validated using Group B. In order to avoid non-disease related bias, the Group A patients were age and gender matched (DLB: 76 ± 4 years, 12 males, 12 females; AD, 76 ± 5 years, 12 males, 12 females). Group B contained the remaining patients (23 DLB-patients; age: 76 ± 9 years, 8 females and 73 AD-patients; age 74 ± 9 years, 58 females). Additionally, nine Parkinson Disease patients (70 ± 7 years, 4 females) were included in the study (Table 1, Supplementary Table 6). Three patient samples (two AD and one DLB) were excluded from the analysis after initial assessment, as they appeared to be strong outliers, likely due to failed sample storage or preparation.

### Sample preparation

Experimental design and approaches were permitted by and conducted at Department of Medical Biochemistry and Biophysics, Karolinska Institutet, Sweden.

#### Intact proteome

Serum samples were digested with trypsin using Protease MAX^TM^ Surfactant, Trypsin Enhancer (Promega) and urea according to a modified protocol as previously described^47^. 10 μg of total protein per sample were reduced with 20 mM dithiothreitol for 30 min at 56 °C and alkylated with 66 mM iodoacetamide for 30 min in the dark. Trypsin was added at a ratio of 1:30 (enzyme:protein) and the proteins were digested at 37° overnight. Tryptic peptides were desalted using C18 StageTips (Thermo Scientific), dried in a SpeedVac and resuspended in 0.1% formic acid and 1% acetonitrile.

#### MG-enrichment

Polyclonal IgGs and associated proteins were enriched from blood serum using Melon Gel IgG Spin Purification Kit according to the protocol provided by the manufacturer (Thermo Scientific). Briefly, 40 μL aliquots of serum were diluted with Melon Gel Purification Buffer (1:10). 500 μL of Melon gel slurry/sample were washed twice with 300 μL purification buffer (30 s at 2,500 g). The samples were added to the Melon Gel columns and incubated at 20 °C for 30 min using end-over-end mixing. The IgG-molecules with associated proteins were collected via centrifugation (60 s at 2,500 g). IgG enrichment was confirmed on a pooled sample of the MG-extracted IgG from all patients using denaturating SDS-PAGE mini gel system (NuPAGE^®^ Bis-Tris Mini Gel, Sigma Aldrich). A human pooled plasma standard (SeraLab) and a human polyclonal IgG standard (Sigma Aldrich) were used as controls (Supplementary Fig. 6). Samples were stored at −80 °C until trypsin digestion, which was performed similar to the proteomics samples described above (but excluding the first precipitation step).

Ready peptide mixtures were kept at 10 °C and injected onto a chromatographic column in 1 μg aliquots.

### Liquid chromatography - tandem mass spectrometry (nLC-MS/MS) analysis

All samples were analyzed in singlets (running order is provided in Supplementary Table 6).

#### Intact proteome

A reversed phase liquid chromatography system Easy-nLC II coupled in-line with a Q Exactive Plus Orbitrap mass spectrometer (both - Thermo Fisher Scientific) was used. The chromatographic separation was achieved on a 10 cm column in-house packed with 3 μm C18-AQ ReproSil-Pur^®^ (Dr. Maisch GmbH, Ammerbuch-Entringen, Germany) using a 90 min elution gradient from 5–35% of solution B (98% acetonitrile).

Positive mode electrospray ionization was used. The mass spectra were acquired in data-dependent acquisition (DDA) mode. A survey mass spectrum in the range of m/z 300–1650 obtained at a nominal resolution of 70,000 was followed by the selection for MS/MS of the top ten most abundant precursor ions. MS/MS was performed using higher energy collisional dissociation (HCD) with normalized collision energy of 26 and detection at a resolution of 17,500.

#### MG-enriched proteome

A nano-liquid chromatography system Ultimate 3000 connected in-line to a Fusion Orbitrap mass spectrometer (both - ThermoFisher Scientific) was used. Reversed phase LC-separation of the peptides was performed on a 15 cm long EASY spray column (PepMap, C18, 3 μm, 100 Å). The chromatographic separation was achieved using a gradient solvent system containing (A) water with 2% acetonitrile and 0.1% formic acid and (B) acetonitrile with 2% water and 0.1% formic acid. The gradient was set up as follows: 1–30% (B) in 94 min, 31–95% (B) in 5 min, 95% (B) for 8 min and 1% (B) for 10 min. The flow rate was set at 300 nL/min. The mass spectrometer was operating in the positive DDA mode. A survey mass spectrum was acquired in the range of m/z 300–1700 with a nominal resolution of 120,000 (AGC target of 4.0e5 with a maximum injection time of 50 ms). Precursor ion selection was performed in the “top speed” mode of the charge states from 2 to 7, with the most intense precursor priority and with a minimum intensity of 50,000. Dynamic exclusion duration was set as 120 s. Up to five precursor ions were selected for MS/MS, which was performed for each precursor with both HCD (collision energy: 27%, resolution 15,000, AGC target 5.0e4, maximum injection time 200 ms) and electron transfer dissociation (ETD; “collision energy”: 40%, resolution 15,000, AGC target 5.0e4, maximum injection time 200 ms).

### Protein and peptide identification and quantification

#### Database matching

All MS/MS spectra from MG-extraction experiments were firstly searched against the human reference proteome (89,027 UniProt protein sequences, February 2014). Morpheus (v.165) was used as a search engine, allowing up to two missed tryptic cleavages, with 10 ppm and 20 ppm mass tolerances for precursor and fragment peaks, respectively. Carbamidomethylation of cysteine was set as a fixed modification; variable modifications included oxidation of methionine, deamidation of asparagine and glutamine, as well as acetylation of protein N-terminus. MS/MS spectra assigned to peptide sequences with <1% FDR were then excluded from the dataset. The remaining data underwent *de novo* sequencing.

#### De novo sequencing

The remaining unassigned spectra were pair-wisely (HCD-ETD) submitted to pNovo + (v.1.3)^26^. Precursor mass range was limited to between 700 to 4000 Da, oxidized methionine was considered as an independent residue, and mass tolerance was set at 5 ppm for precursors and 15 ppm for fragments in MS/MS. All candidate sequences were filtered by the criteria of full backbone coverage by the fragments. Three top-scoring peptide sequences were kept as candidates. The *de novo* sequencing process was repeated for each HCD-ETD pair with the precursor mass shifted +1.003 and −1.003 Da, in order to correct potential errors in monoisotopic mass assignment. Therefore, up to nine top sequence candidates were generated for each HCD-ETD pair. These candidates were homology-searched against the human UniProt protein database (89,027 protein sequences, February 2014) using BLASTp. Two scores were given for each *de novo* peptide. The *de novo* scores (as reported by pNovo+) ranged between 30 to 147 (mean ± S.D: 38 ± 17), and the corresponding BLAST scores ranged between 31 to 125 (mean ± S.D: 52 ± 14). BLAST scores were calculated combining three elements, 1) number of identical residues, 2) number of positively scored residues and 3) BitScores^48^. Notably, both the de novo score and the BLAST score are positively related to the sequence length. *De novo* sequenced peptides and the corresponding scores are given in Supplementary Table 7. Assignment of CDR and FR regions were based on Uniprot information and by using the VBASE sequence directory (Tomlinson *et al*., MRC Centre for Protein Engineering, http://www2.mrc-lmb.cam.ac.uk/vbase/alignments2.php). Since leucine (Leu/L) and isoleucine (Iso/I) were difficult to distinguish in *de novo* sequencing, all isoleucine residues (I) in the protein sequence database were converted to leucine (L). The match with the highest BLAST score was reported as the final sequence for a given HCD-ETD spectral pair.

The UniProt human protein database and all obtained *de novo* sequences were merged in a new SpotLight database, on which a second database search was then performed as described below and followed by quantification.

#### Quantification

Raw mass spectrometry data were processed through the DeMix-Q workflow^23^,^24^, in which MS/MS spectra were matched against the SpotLight database. Morpheus search engine was used with the same parameters as described above. MS/MS identifications with <1% FDR were assigned to chromatographic features that were assembled from MS1 spectra using OpenMS4^49^. Maps of chromatographic features from individual LC-MS experiments were aligned and clustered into a consensus map, which contains the information of *m/z*, retention time, charge states, abundances and possible peptide sequences. If a consensus feature was associated with more than one peptide sequence or different sequences in different LC-MS/MS runs, only the most common association was kept. Peptide abundances were reported as the summed integrals of ion currents from all charge states. Proteins identifications were reported by applying the rule of maximum parsimony, and the *de novo* sequences were assigned to the proteins they matched in the BLAST search. Protein abundances were calculated by averaging the abundances of three most abundant constituent peptides. Only proteins from which we could identify at least two unique peptides per protein were used. Only the proteins and peptides that could be found and quantified in at least 50% of either all DLB- or all AD-patients were included in the output. The abundances of MG-enriched IgG peptides were re-normalized such that their total abundance in all samples was the same, both for the intact and MG-enriched proteome. The non-IgG peptides were normalized separately in the same way. On the protein level, all protein abundances were normalized to add to 100% in total in the sample.

### Fc-glycan profiling

Fc-glycan profiling was performed as previously described^21^,^50^. Briefly, IgG Fc-glycopeptides were identified and quantified in the LC-MS/MS datasets (from the raw files generated in the MG-enriched proteome analyses that were acquired on the Ultimate 3000 LC system connected on-line to the Fusion Orbitrap mass spectrometer, as described in the nLC-MS/MS section). Glycopeptides were identified by their characteristic retention times (as determined by IgG standard) and accurate monoisotopic masses (within <10 ppm from the theoretical values) of doubly and triply charged ions (IgG_1_: EEQYNSTYR, IgG_2/(3)_: EEQFNSTFR, IgG_3/4_: EEQYNSTFR/EEQFNSTYR) as well as of triply and quadruply charged ions (IgG_1_: TKPREEQYNSTYR, IgG_2/(3)_: TKPREEQFNSTFR and IgG_3/4_: TKPREEQYNSTFR/TKPREEQFNSTYR). Abundances of IgG_1_, IgG_2/(3)_ and IgG_3/4_ glycopeptides were normalized by their respective total content. The list of the glycoforms that were screened for, their relative distribution and abundance differences between the two patient cohorts is given in Supplementary Table 8.

### Statistics

Univariate statistical analysis was performed using two-tailed Student’s t-test with equal or unequal variance depending upon the F-test. P-values were adjusted with Bonferroni correction for the number of tested variables. ROC-curve analysis and linear regression tests were performed using PRISM (GraphPad Software, CA, USA). Principal component analysis (PCA) and orthogonal projections to latent structures discriminate analysis (OPLS-DA) was performed using SIMCA 14.0 (Umetrics, Umeå, Sweden) following mean centering, log scaling and UV scaling. Model performance was reported as the cumulative correlation R^2^X[cum], and predictive performance – as Q^2^[cum] based on seven-fold cross validation. OPLS-DA models were further validated using ANalysis Of VAriance testing of Cross-Validated predictive residuals (CV-ANOVA), cross validated scores t (tCV) and predicted scores t (tPS).

## Additional Information

**How to cite this article**: Lundström, S. L. *et al*. SpotLight Proteomics: uncovering the hidden blood proteome improves diagnostic power of proteomics. *Sci. Rep.* **7**, 41929; doi: 10.1038/srep41929 (2017).

**Publisher's note:** Springer Nature remains neutral with regard to jurisdictional claims in published maps and institutional affiliations.

## Supplementary Material

Supplementary Information

Supplementary Table 1

Supplementary Table 3

Supplementary Table 4

Supplementary Table 6

Supplementary Table 7

## Figures and Tables

**Figure 1:**
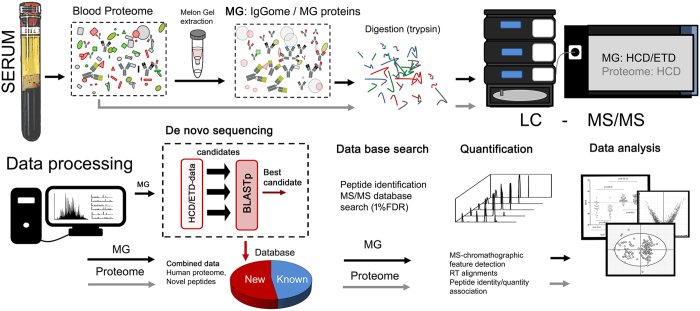
Approach overview. Schematic overview of the SpotLight approach. In short, blood sample is digested and analyzed by LC-MS/MS. In parallel, the same sample is enriched using Melon Gel and digested, with *de novo* sequencing of the tryptic fragments. The novel sequence candidates are BLASTed and thus either assigned to known proteins or IgG, or discarded. The assigned sequences are added to the convention protein sequence database, and all MS/MS data are searched in this expanded database. The sequences with <1 FDR are then quantified, and multivariate analysis is performed on both the peptide level as well as the protein level.

**Table 1:** Patient characteristics and patient groups included in the study.

Cohort	Type	n^A^	Female	Age^B^	MMSE^C^ baseline	Annual decline MMSE
GroupA	DLB^D^	24	50%	76 ± 4	24 ± 3	3 ± 3
	AD^E^	24	50%	76 ± 5	25 ± 2	3 ± 2
GroupB	DLB	23	35%	76 ± 9	22 ± 4	3 ± 3
	AD	73	79%	74 ± 9	23 ± 2	3 ± 2
PD-patients	PD	9	45%	70 ± 7	26 ± 2	2 ± 2

For individual clinical information see Supplementary Table 6. ^A^Number, ^B^Years, ^C^Mini Mental State Examination, ^D^Dementia with Lewy Bodies, ^E^Alzheimer’s disease.

**Table 2:** Proteins and peptides of particular interest with different abundances in AD and DLB samples.

Domain	Protein/Peptide	ID/origin	Peptides	DLB^A^ Mean ± STD^C^	AD^B^ Mean ± STD	p-value	Corrected
Proteome	Serum amyloid P-component	SAMP_HUMAN	6	99 ± 56	167 ± 62	1.9E-09	9.5E-06
	Transthyretin	TTHY_HUMAN	8	8191 ± 1514	5603 ± 2649	9.9E-12	4.9E-08
	Apolipoprotein D	C9JF17_HUMAN	5	1358 ± 499	1793 ± 494	2.2E-06	1.1E-02
	Multiple PDZ domain protein	B7ZB24_HUMAN	2	545 ± 273	323 ± 239	2.4E-06	1.2E-02
Melon Gel proteins	Properdin	PROP_HUMAN	6	983 ± 665	1629 ± 663	1.9E-07	9.4E-04
	Plasma kallikrein	KLKB1_HUMAN	27	5869 ± 2789	8043 ± 210	1.1E-05	5.2E-02^D^
	Complement C1q subcomponent subunit B	C1QB_HUMAN	2	461 ± 265	808 ± 558	1.3E-06	6.3E-03
*De novo* sequences	QTGPTAGWNLPGPVSVGFK	TGPTAGRDLLLPSPVS/F2Z3L0_HUMAN	1	20 ± 21	43 ± 23	9.5E-08	4.7E-04
	GTAGWNLDSPRLYGGK	NLDSPKLY/SEM6D_HUMAN	1	73 ± 46	108 ± 37	4.0E-06	2.0E-02
	GDGVAEQYADSYAQYCNPR	AESYAQYVHNLCN/F5H702_HUMAN	1	90 ± 81	196 ± 70	3.0E-13	1.5E-09
	GDGVEAMNEQAHAQYCNPR	GVGALEQEHAQY/F8W6 × 8_HUMAN	1	18 ± 21	53 ± 25	5.8E-14	2.9E-10
	PGSVFPLADVGGK (MG)	PDSVFPLEGASDADVG/PCDA6_HUMAN	1	17 ± 29	50 ± 53	3.8E-06	1.9E-02
	PGSVFPLADVGGK (proteome)	PDSVFPLEGASDADVG/PCDA6_HUMAN	1	2 ± 2	12 ± 13	6.4E-05	2.7E-01^D^
	NTLYLQMGNSLR	NTLFLQMDSLR/FR3^E^/HV311_HUMAN	1	231 ± 154	447 ± 192	3.8E-10	1.9E-06
Other	SSQSVLYSSNNK	CDR1^F^/KV401_HUMAN	1	42 ± 54	100 ± 94	5.7E-06	2.8E-02

Mean and standard deviations are given in ppm (total relative abundance in each domain = 1,000,000). P-values are given with and without Bonferroni correction. For full list of peptides and proteins see Supplementary Table 4. ^A^Dementia with Lewy Bodies, ^B^Alzheimer’s disease, ^C^Standard Deviation, ^D^Reaches significance when PD patients are included, Supplementary Table 4 ^E^Framework, ^F^Complement determining region.
