# Supplementary material for: SpotLight Proteomics: uncovering the hidden blood proteome improves diagnostic power of proteomics
Source: Sci Rep. 2017 Feb 7;7:41929. doi: 10.1038/srep41929 (PMC5294601; doi:10.1038/srep41929)
Supplement: Supplementary Information [file srep41929-s1.pdf]

Supplementary information

SpotLight Proteomics: uncovering the hidden blood proteome improves diagnostic power of proteomics

Susanna L. Lundström, Bo Zhang, Dorothea Rutishauser, Dag Aarsland, Roman A. Zubarev

Supplementary Table 1: Excel file

Supplementary Table 2: Page 2

Supplementary Table 3: Excel file

Supplementary Table 4: Excel file

Supplementary Table 5: Page 3

Supplementary Table 6: Excel file

Supplementary Table 7: Excel file

Supplementary Table 8: Page 4

Supplementary Figure 1: Page 5

Supplementary Figure 2: Page 6

Supplementary Figure 3: Page 7

Supplementary Figure 4: Page 8

Supplementary Figure 5: Page 9

Supplementary Figure 6: Page 10

References: Page 11

Supplementary Table 2. Predictive power of proteins and peptides derived from the MG-extraction- and proteomics approach. Mean and standard deviations of the Predicted scores (tPS) of the Group B patients (treated as unknowns) and nine PD patients (treated as unknowns) in the OPLS-DA models based on differences in the AD- and DLB-Group A patients.

Content	Sample Set	Domain	DLB[A] n=23	AD[B] n=73	PD[C] n=9	AD vs DLB p-value	AD vs [DLB and PD] p-value	ROC[D] AUC[E]	n
Proteins	Full Model		-1.3±2.4	1.2±1.5	-2.1±1.9	8.5E-05	2.3E-07	0.85	237
	Melon Gel		-1.8±2.5	0.2±2.3	-0.9±3.1	6.8E-04	9.9E-04	0.66	81
	Proteome		-0.9±1.9	1.2±1.3	-1.4±1.2	3.9E-05	1.0E-10	0.85	156
Peptides	Full Model		-7.0±9.0	6.6±5.3	-7.3±4.9	2.1E-07	2.4E-11	0.94	4708
	Melon Gel	IgGome, MG proteins	-8.3±9.3	4.7±7.6	-3.7±6.6	1.3E-09	4.9E-10	0.84	2129
		IgGome	-2.5±4.7	2.7±3.6	-1.4±2.8	2.4E-07	2.6E-08	0.82	646
		MG proteins	-8.0±8.4	3.9±6.8	-3.4±6.4	4.8E-10	3.1E-10	0.84	1431
		Fc-glycopeptides	-0.7±1.8	0.9±2.1	-2.3±2.8	7.4E-01	5.2E-01	0.53	52
	Proteome	Proteome	-1.9±5.0	4.6±4.0	-7.0±2.6	6.3E-09	7.1E-14	0.88	2579
New sequences	Full Model		-4.7±7.5	4.9±4.2	-6.7±4.3	3.3E-06	8.1E-10	0.90	1997
	Melon Gel	IgGome, MG proteins	-4.8±7.6	4.5±5.6	-2.6±4.4	7.1E-09	5.6E-10	0.84	1213
		IgGome	-2.1±4.4	2.5±3.3	-1.3±2.2	4.3E-07	3.0E-08	0.82	507
		MG proteins	-4.2±6.7	3.9±4.8	-2.0±4.3	8.0E-06	9.2E-10	0.84	706
	Proteome	Proteome	-1.2±4.2	3.0±2.5	-5.1±2.1	8.9E-05	2.6E-08	0.85	784
Known sequences	Full Model		-4.5±5.9	4.6±4.3	-3.1±4.1	3.7E-12	5.2E-14	0.92	2659
	Melon Gel	IgGome, MG proteins	-5.8±7.1	2.6±6.4	1.5±6.9	4.9E-07	1.2E-06	0.76	864
		IgGome	-1.2±2.0	1.1±1.8	-0.3±2.0	1.2E-06	1.3E-06	0.77	139
		MG proteins	-3.9±5.7	2.8±4.1	-0.1±4.9	1.2E-05	6.6E-06	0.78	725
	Proteome	Proteome	-1.1±3.6	3.6±3.9	-4.0±2.9	2.0E-06	9.0E-10	0.84	1795

[A]Dementia with Lewy Bodies, [B]Alzheimer's disease, [C]Parkinson's Disease, Standard Deviation, [D]Receiver operating characteristic, [E]Area Under Curve

Supplementary Table 5. Significant differences in CV-scores between the DLB and AD patients split up according to ApoE-genotype (E2-E3 and E3-E3 versus E3-E4 and E4-E4).

Content	Sample set	Domain	Differences ApoE AD vs DLB+PD		(E2-E3 or E3-E3) vs (E3-E4 or E4-E4)	
			E2-E3 or E3-E3	E3-E4 or E4-E4	AD	DLB+PD
Proteins	Full Model		1.6E-05	3.8E-16	1.4E-01	1.5E-02
	Melon Gel		8.6E-04	5.7E-08	6.8E-01	4.9E-01
	Proteome		5.9E-04	7.2E-18	1.0E-01	8.8E-03
Peptides	Full Model		3.4E-07	1.0E-13	5.7E-02	7.5E-02
	Melon Gel	IgGome + MG proteins	2.0E-05	2.5E-09	7.8E-03	5.0E-01
		IgGome	9.5E-04	2.7E-11	2.5E-02	3.3E-01
		MG proteins	5.6E-05	3.1E-08	1.5E-01	3.4E-01
		Fc-glycans	4.5E-02	1.8E-03	1.4E-02	7.5E-01
	Proteome		1.0E-05	2.5E-20	2.0E-02	2.2E-01
New sequenes	Full Model		1.9E-05	3.3E-18	1.3E-03	1.2E-01
	Melon Gel	IgGome + MG proteins	1.3E-04	3.6E-13	2.9E-02	1.7E-01
		IgGome	2.1E-03	1.6E-07	8.2E-03	5.1E-01
		MG proteins	4.9E-05	2.5E-13	2.5E-02	1.7E-01
	Proteome		4.7E-04	1.1E-16	1.4E-03	5.4E-01
Known sequences	Full Model		2.9E-06	1.4E-13	5.3E-02	2.1E-01
	Melon Gel	IgGome + MG proteins	8.4E-05	2.9E-08	8.4E-02	6.2E-01
		IgGome	1.3E-03	1.1E-09	2.1E-02	5.2E-01
		MG proteins	7.6E-05	1.5E-10	1.3E-01	6.6E-01
	Proteome		1.0E-05	1.9E-17	7.6E-02	2.9E-01

Supplementary Table 8. Fc-glycan distribution in the AD and DLB patients. Given p-values are not Bonferroni corrected. Due to polymorphism IgG_3 can be found as either EEQYNSTFR or EEQFNSTFR, (the latter particularly common in caucasians)[1]. Also note that peptides EEQYNSTFR (IgG_3) and EEQFNSTYR (IgG_4) comes at the same retention time and has the same mass (i.e. we can not distinguish them). Note that this likely is the main reason why the variation in particularly $IgG_{3/4}$ is quite high. (Since IgG_2 is more abundant than IgG_3 the $IgG_{2/3}$ values are less affected).

		IgG_1 EEQYNSTYR TKPREEQYNSTYR			$IgG_{2/3}$ EEQFNSTFR TKPREEQFNSTFR			$IgG_{4/3}$ EEQYNSTFR or EEQFNSTYR TKPREEQYNSTFR or TKPREEQFNSTYR		
Suggested structure[a]	Composition[b]	AD	DLB	p-value	AD	DLB	p-value	AD	DLB	p-value
A2	HexNAc(4)Hex(3)	1.5±0.9	1.5±0.9	0.9	2.5±2.0	2.5±2.1	1.0	0.3±0.4	0.6±0.8	0.1
FA1	dHex(1)HexNAc(3)Hex(3)	0.3±0.1	0.3±0.2	0.5	0.9±0.4	1.1±0.6	0.1	0.5±3.8	0.2±0.2	0.4
FA2	dHex(1)HexNAc(4)Hex(3)	23.6±6.9	23.3±6.9	0.8	35.7±7.7	37.2±7.2	0.3	31.4±10.1	32.0±10.3	0.7
A2G1	HexNAc(4)Hex(4)	2.0±1.3	2.0±1.1	0.7	2.5±2.8	1.8±1.3	**0.04**	-	-	-
FA1G1	dHex(1)HexNAc(3)Hex(4)	0.1±0.1	0.1±0.1	0.4	0.2±0.1	0.3±0.2	**0.02**	0.04±0.1	0.1±0.1	0.2
FA2G1	dHex(1)HexNAc(4)Hex(4)	26.4±4.7	25.8±4.6	0.5	23.8±4.8	23.8±4.4	1.0	18.3±5.8	19.2±5.4	0.4
FA2G2	dHex(1)HexNAc(4)Hex(5)	10.4±3.5	10.1±3.4	0.7	6.7±2.4	6.6±2.2	0.8	14.1±16.2	10.7±11.6	0.1
A2G2	HexNAc(4)Hex(5)	0.8±0.6	0.8±0.6	0.9	1.0±1.0	0.9±0.6	0.5	-	-	-
FA2G1S	dHex(1)HexNAc(4)Hex(4)NeuAc(1)	1.4±1.7	1.3±0.6	0.5	2.8±1.3	3.1±1.0	0.1	1.9±1.1	1.9±0.9	0.9
FA1G1S1	dHex(1)HexNAc(3)Hex(4)NeuA(1)	0.1±0.1	0.1±0.1	0.8	0.4±0.3	0.8±1.0	**0.003**	0.3±0.6	0.5±0.7	0.3
FA2G2S1	dHex(1)HexNAc(4)Hex(5)NeuAc(1)	5.0±1.9	5.6±2.2	0.1	3.0±1.6	3.1±1.5	0.8	4.2±2.8	3.9±2.4	0.6
FA2G2S2	dHex(1)HexNAc(4)Hex(5)NeuAc(2)	0.4±0.7	0.3±0.3	0.2	0.1±0.3	0.02±0.02	0.2	0.7±1.9	0.9±1.9	0.6
A2B	HexNAc(5)Hex(3)	0.8±0.5	0.9±0.6	0.3	0.5±0.3	0.4±0.2	0.2	2.8±4.4	7.0±10.3	**0.01**
FA2B	dHex(1)HexNAc(5)Hex(3)	11.8±4.3	12.3±4.7	0.5	13.9±4.1	12.9±3.0	0.1	16.5±5.8	14.9±6.3	0.1
FA2BG1	dHex(1)HexNAc(5)Hex(4)	13.5±3.8	13.7±3.6	0.7	5.8±2.4	5.2±1.9	0.1	7.6±2.8	6.2±3.3	**0.01**
FA2BG2	dHex(1)HexNAc(5)Hex(5)	1.6±1.0	1.5±0.7	0.5	0.2±0.6	0.2±0.3	0.3	0.9±0.7	1.0±0.8	0.5
FA2BG1S1	dHex(1)HexNAc(5)Hex(4)NeuAc(1)	0.2±0.1	0.2±0.2	0.8	0.1±0.2	0.05±0.04	0.3	0.2±0.3	0.8±1.5	**0.01**
FA2BG2S1	dHex(1)HexNAc(5)Hex(5)NeuAc(1)	0.1±0.1	0.1±0.2	0.8	0.1±0.1	0.1±0.1	0.3	0.1±0.1	0.4±1.1	0.1
FA2BG2S2	dHex(1)HexNAc(5)Hex(5)NeuAc(2)	-	-	-	-	-	-	-	-	-

[a]Glycan nomenclature is described by Royle et al [2]. A2 is defined as the core heptasaccharide moiety which can be further substituted by fucose (F), galactose (G) and sialic acid (S). B indicates a bisecting N-acetyl-glucoseamine unit linked to A2. [b] Sugar composition for respective structure; N-acetyl-glucoseamine (HexNAc), Hexose (Hex), Deoxy hexose (dHex) Neuraminic acid (NeuAc).

Supplementary Figure 1. Distributions between the peptides identified by database search and de novo sequenced peptides. (A) The distribution between total number of quantified peptides identified by database search (n=1310) and de novo sequenced peptides (n=1709). Median abundances (in log10 scale) are 6.08 for known peptides and 6.08 for de novo peptides. As expected, most of the highly abundant peptides are known. (**B**) The distribution between filtered peptides quantified in ≥50% of respective cohort. 864 peptides were identified by database search and 1213 by de novo sequencing. Median abundances (in log10 scale) are 6.97 for known peptides and 6.33 for de novo peptides. Median abundances (in log10 scale) are 6.47 for known peptides and 6.33 for de novo peptides. As expected, most of the highly abundant peptides are known.

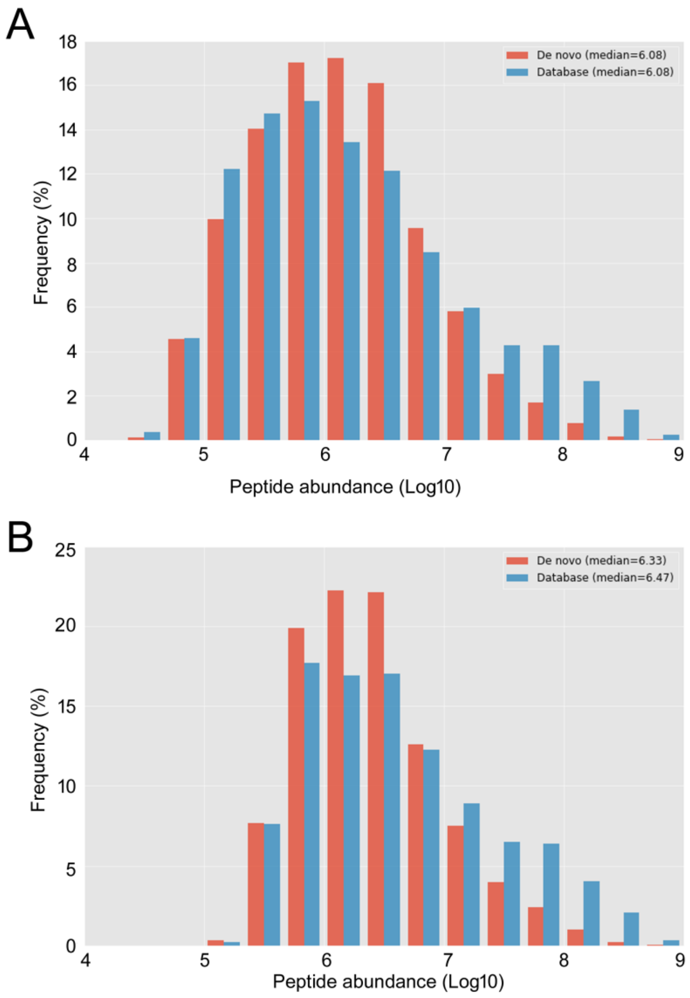

Supplementary Figure 2. Overview and correlation between the data sets. (A) Distributions and numbers of known and de novo sequenced peptides derived from the Melon Gel and Proteomics approaches. Peptides were grouped according to conserved and variable IgG regions as well as peptides with no sequence homology to IgG (i.e. other peptides). Variable peptides are divided into heavy variable (HV), kappa variable (KV) and lamba variable (LV) peptides, respectively. **(B)** The correlation between the average reference abundance of IgGome peptides quantified in both the proteome and Melon Gel enriched fractions. **(C)** The same correlation for peptides quantified in both matrixes but with no IgG sequence homology.

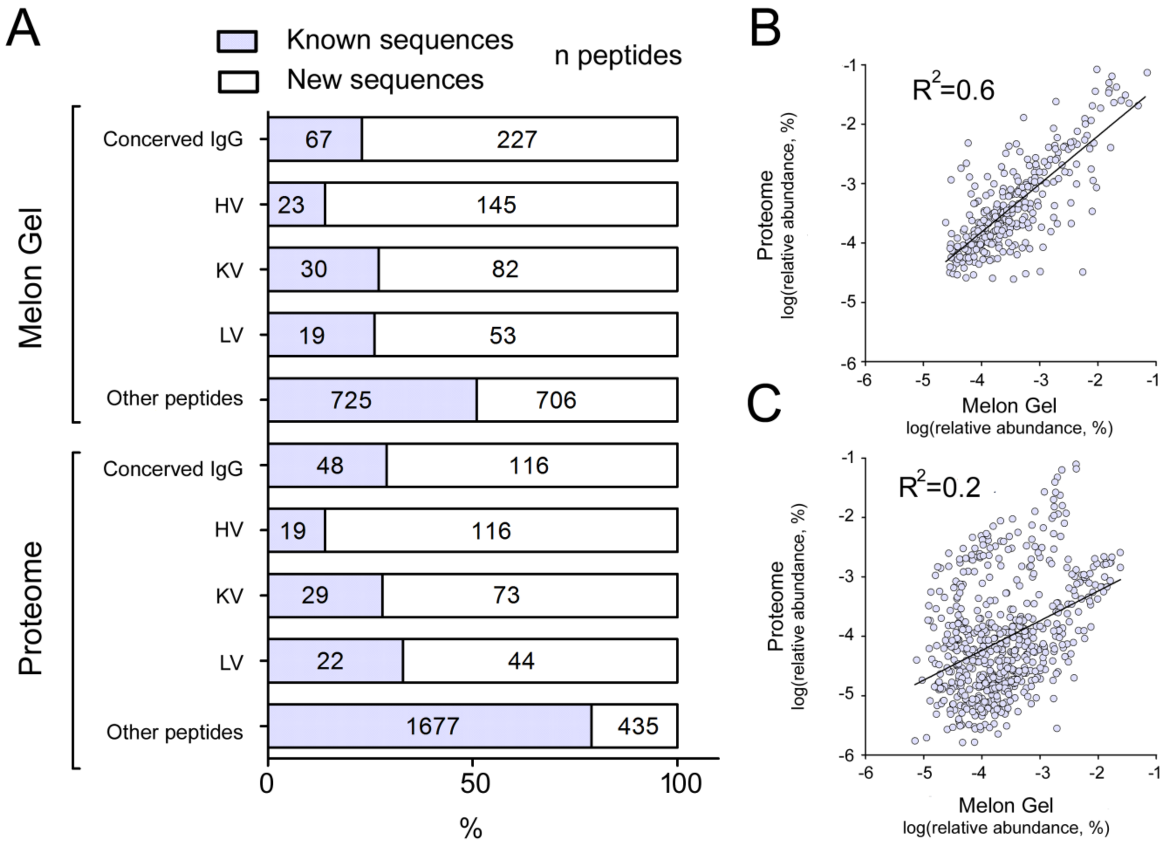

Supplementary Figure 3. PCA scores plot based on the complete data set and all patients (R^2=0.51, Q^2=0.21, 23 components). The figure shows component 3 and component 5 for which the best separation between the groups was observed.

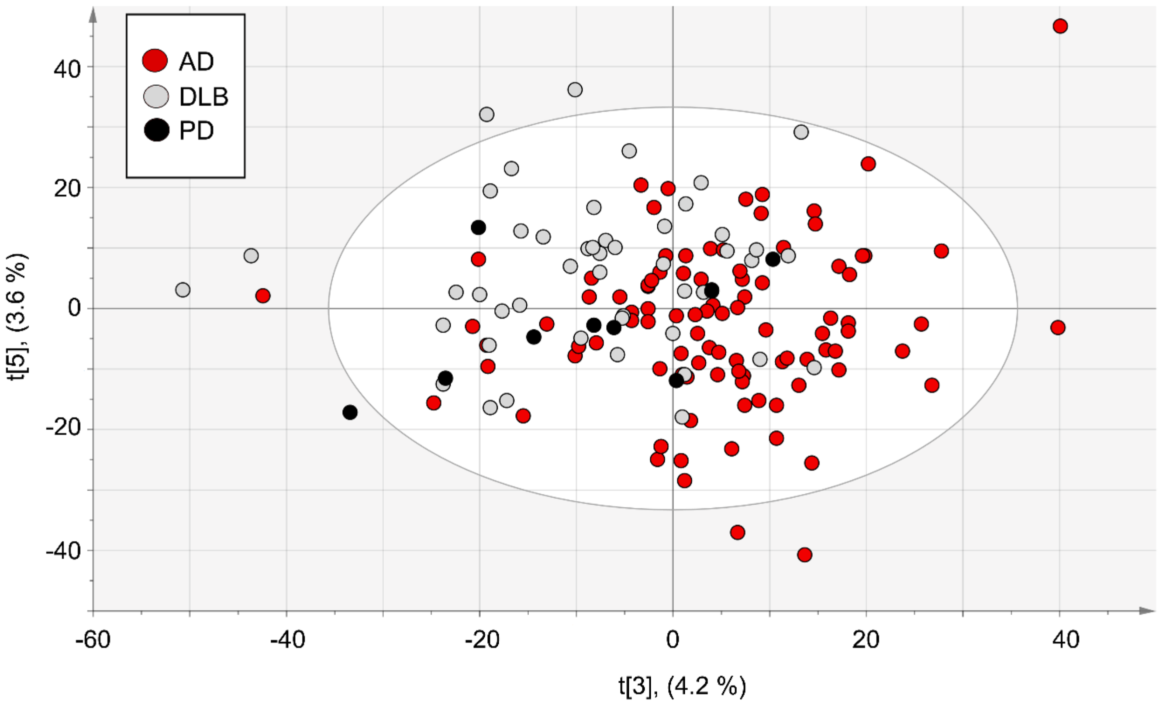

Supplementary Figure 4. Peptides derived from the FR3 region of HV3. The model is the same as in Figure 3B but with only the FR3-HV3 peptides shown. The HV3 chain FR3 peptide NTLYLQMGNSLR (Table 2) is specific for the AD cohort. In contrast, all 11 other peptides from this region correlate with the DLB cohort. Only the AD peptide remains significant following Bonferroni Correction (p=1.9E-06).

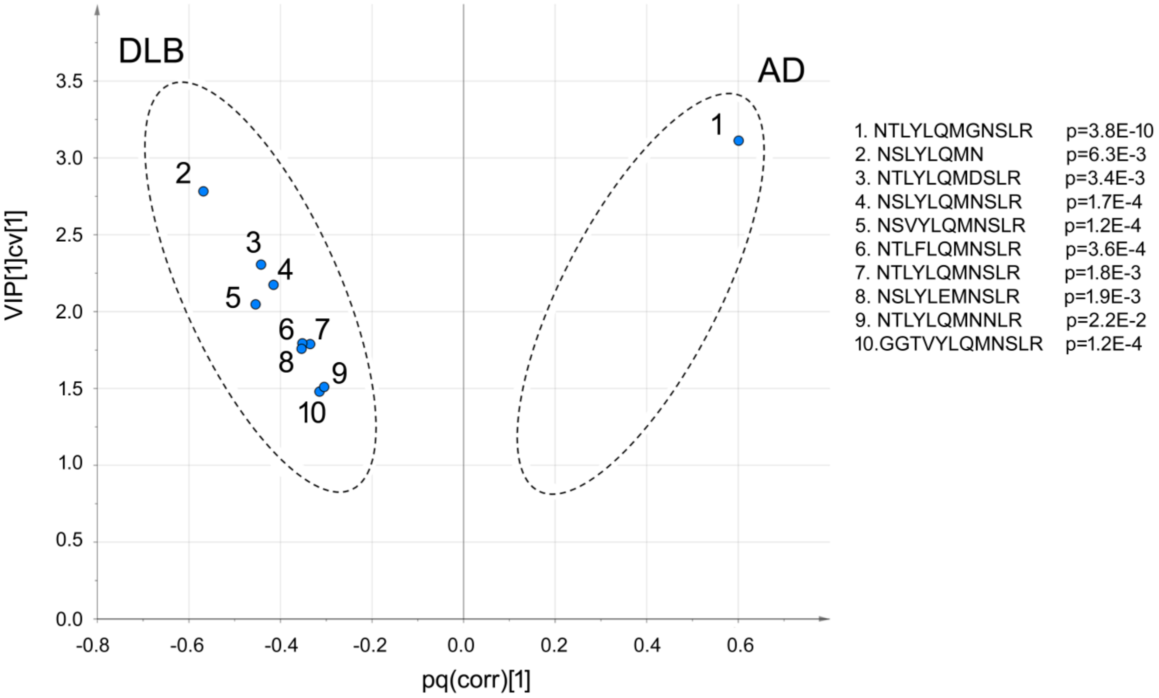

Supplementary Figure 5. Examples of the predictability of some of the proteins and peptides in the intact and Melon Gel proteomes

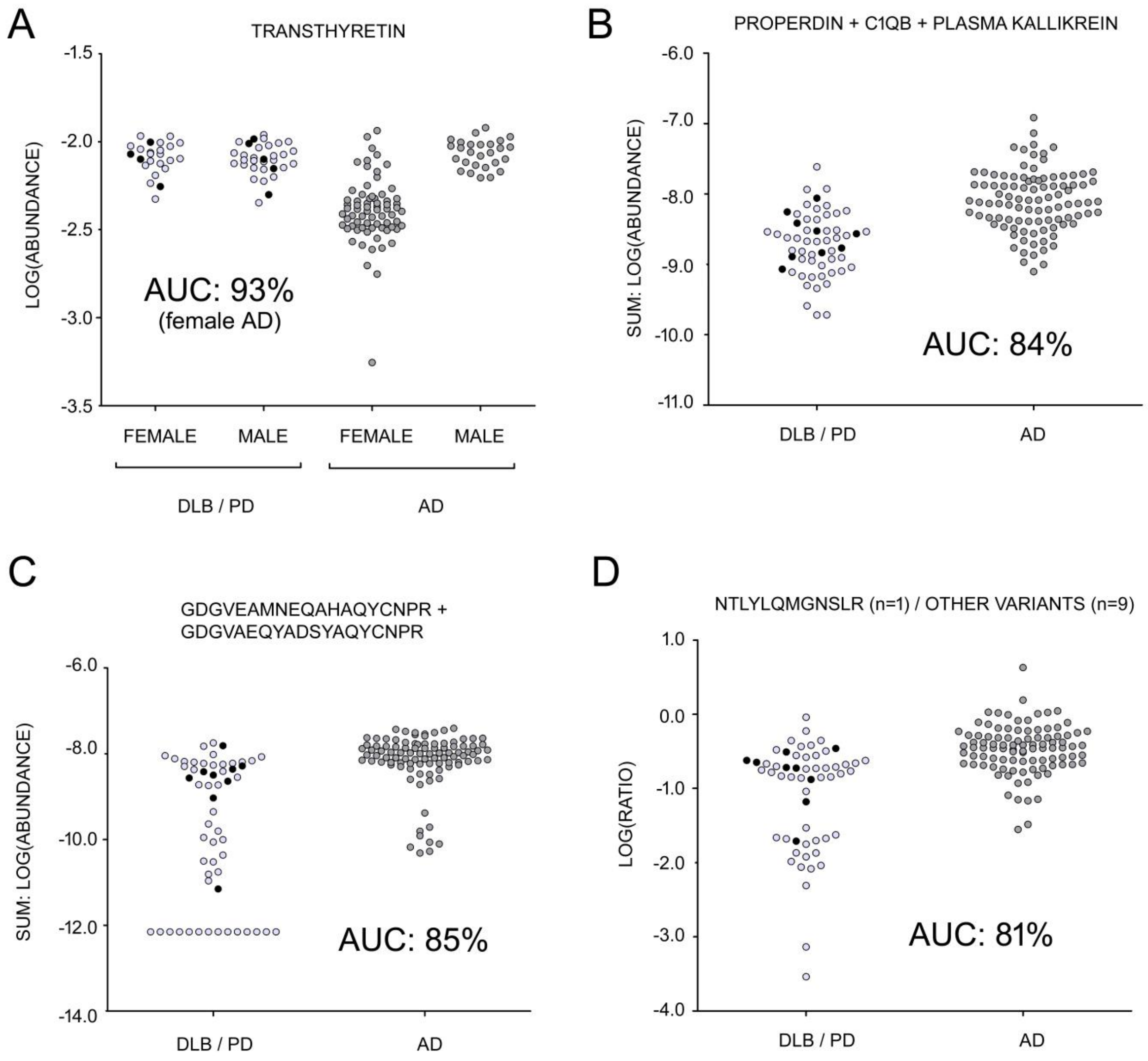

Supplementary Figure 6. SDS-PAGE Gel analysis of human plasma, human polyclonal IgG and pooled MG extracted IgG from the patients. In addition to IgG heavy and light chains, particularly serrotransferrin is consistently found in relatively high abundance following the IgG extraction in the MG enriched samples.

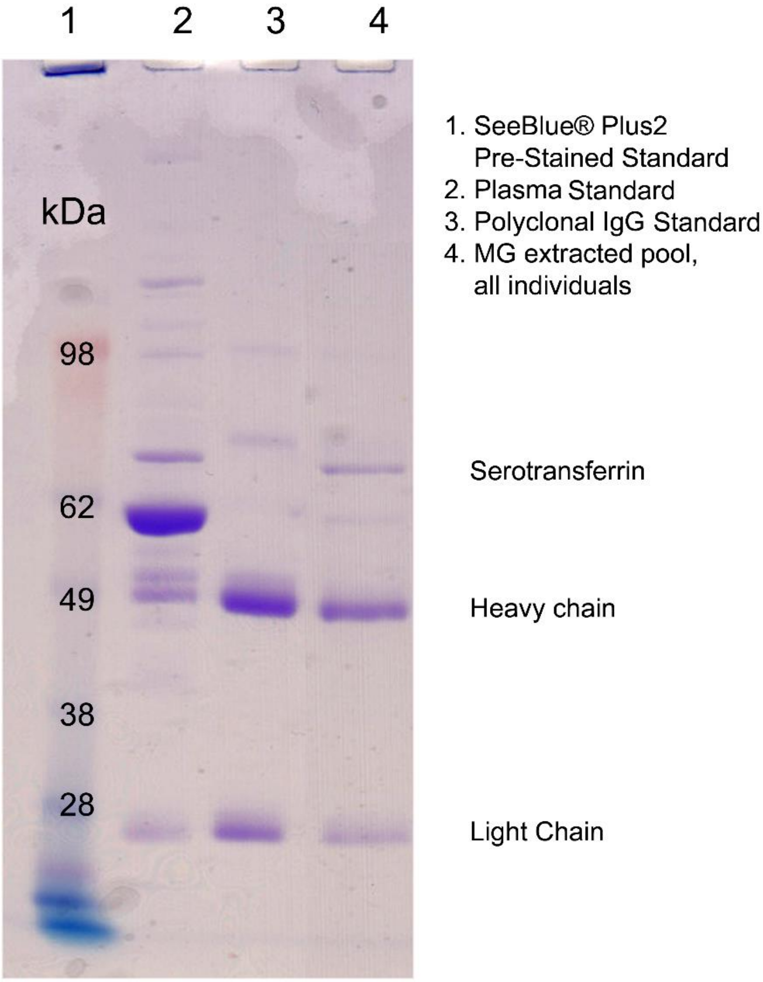

References

1. Balbin, M., Grubb, A., de Lange, G.G. & Grubb, R. DNA sequences specific for Caucasian G3m(b) and (g) allotypes: allotyping at the genomic level. *Immunogenetics* **39**, 187-193 (1994).
2. Royle, L.*, et al.* HPLC-based analysis of serum N-glycans on a 96-well plate platform with dedicated database software. *Anal Biochem* **376**, 1-12 (2008).
